# Bio-based nanomaterials in drug delivery: An updated review

**DOI:** 10.1016/j.pscia.2026.100126

**Published:** 2026-05-22

**Authors:** Great Iruoghene Edo, Ali B.M. Ali, Michael Chukwuma Okolie, Joshua Othuke Orogu, Kugbere Emumejaye, Ephraim Evi Alex Oghroro, Agatha Ngukuran Jikah, Emad Yousif, Ufuoma Augustina Igbuku, Joseph Oghenewogaga Owheruo, Arthur Efeoghene Athan Essaghah, Dina S. Ahmed, Raghda S. Makia, Huzaifa Umar, Ahmed A. Alamiery, Ibiyinka Agboola Fuwape

**Affiliations:** a Department of Chemistry, Faculty of Science, Southern Delta University, Ozoro, Nigeria; b Department of Chemistry, College of Sciences, Al-Nahrain University, Baghdad, Iraq; c Advanced Technical College, University of Warith AlAnbiyaa, Karbala, Iraq; d Department of Food Science and Technology, Faculty of Science, Southern Delta University, Ozoro, Nigeria; e Department of Microbiology, Faculty of Science, Southern Delta University, Ozoro, Nigeria; f Department of Physics, Faculty of Science, Southern Delta University, Ozoro, Nigeria; g Department of Pharmacy, Faculty of Pharmacy, Near East University, Nicosia, Cyprus; h Department of Urban and Regional Planning, Faculty of Environmental Sciences, Southern Delta University, Ozoro, Nigeria; i Department of Chemical and Petroleum Industries Engineering Techniques, Polytechnic College of Engineering Specializations - Baghdad, Middle Technical University, Baghdad, Iraq; j Department of Plant Biotechnology, College of Biotechnology, Al-Nahrain University, Baghdad, Iraq; k Irfan Suat Gunsel Operational Research Institute, Near East University, TRNC Mersin 10, Nicosia, 99138, Turkey; l Al-Ayen Scientific Research Center, Al-Ayen Iraqi University, AUIQ, P.O. Box: 64004, An Nasiriyah, ThiQar, Iraq; m Department of Physics, Michael and Cecilia Ibru University, Agbarha-Otor, Ughelli North, Delta State, Nigeria; n Department of Physics, Federal University of Technology, Akure, Ondo State, Nigeria

**Keywords:** Natural nanomaterials, Targeted therapy, Controlled drug release, Metal-based nanoparticles

## Abstract

Bio-based nanomaterials have emerged as promising drug delivery platforms due to their biocompatibility, biodegradability, and ability to enhance therapeutic performance. Conventional drug delivery systems are often limited by poor drug solubility, rapid degradation, low bioavailability, and nonspecific distribution, particularly for natural bioactive compounds such as polyphenols, alkaloids, and terpenoids. Recent advances in nanotechnology have enabled the development of bio-based nanocarriers capable of improving drug stability, controlled release, intracellular transport, and site-specific delivery. This review provides an updated overview of natural nanomaterials used in drug delivery, including polysaccharide-, protein-, lipid-, and green-synthesized inorganic nanocarriers. Major delivery platforms such as nanoliposomes, micelles, nanosuspensions, solid lipid nanoparticles, and polymeric nanostructures are discussed alongside their mechanisms of action, including passive targeting via the enhanced permeability and retention (EPR) effect, ligand-mediated active targeting, and stimuli-responsive drug release. The review further examines recent applications in cancer therapy, gastrointestinal disorders, delivery of natural bioactive compounds, vaccines, gene therapy, and neurological diseases. In addition to highlighting therapeutic advantages, this review critically discusses current translational challenges, including large-scale manufacturing, batch reproducibility, long-term safety, biodistribution, immunogenicity, and regulatory limitations. A key contribution of this review is the integration of comparative analysis and translational perspectives across major bio-based nanocarrier systems, highlighting the need for standardized evaluation frameworks and scalable production strategies. Future research should focus on reproducible green synthesis methods, quantitative benchmarking, advanced characterization approaches, and clinically relevant validation studies to accelerate the translation of natural nanomaterials into pharmaceutical applications.

## List of abbreviations

**DDS**: Drug Delivery Systems
**NDDS**: Nanotechnology-Based Drug Delivery Systems
**EPR**: Enhanced Permeability and Retention
**RNA**: Ribonucleic Acid
**GI**: Gastrointestinal
**BBB**: Blood–Brain Barrier
**ROS**: Reactive Oxygen Species
**MMPs**: Matrix Metalloproteinases
**PEG**: Polyethylene Glycol
**PLGA**: Poly(lactic-co-glycolic acid)
**Au**: Gold
**Ag**: Silver
**Pt**: Platinum
**Fe**: Iron
**mRNA**: Messenger Ribonucleic Acid
**DNA**: Deoxyribonucleic Acid
**AI**: Artificial Intelligence

## 1 Introduction

**Fig. 1:**
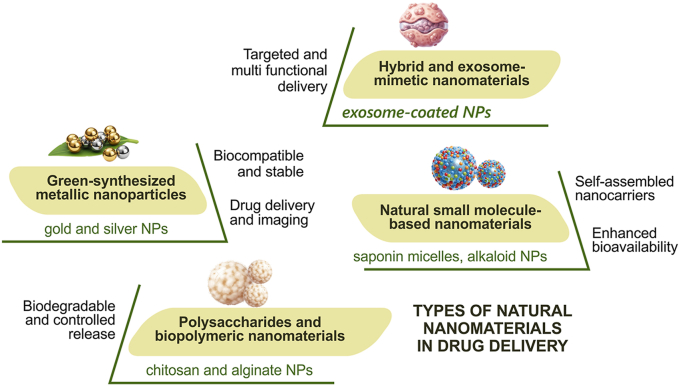
Classification and Key Features of Natural Nanomaterials used in Drug Delivery (This figure was produced using professional graphic software (CorelDRAW, Adobe Illustrator).

Bio-based nanomaterials are nanoscale systems obtained from biological sources or naturally occurring biomolecules that are increasingly investigated as advanced drug delivery platforms due to their biocompatibility, biodegradability, and functional versatility [1,2]. These materials include polysaccharides, proteins, lipids, and other naturally derived compounds that can be engineered to encapsulate, protect, and deliver therapeutic agents with improved efficiency [3]. Compared with many synthetic carriers, bio-based nanomaterials exhibit lower toxicity, reduced immunogenicity, and improved interaction with biological systems, which makes them attractive for pharmaceutical and biomedical applications [4,5]. Related categories of natural nanosystems exist in nanomedicine literature including, naturally occurring nanomaterials (refer to nanoscale biological entities such as exosomes and viruses that inherently exist in biological systems) and bio-based nanomaterials (engineered nanostructures derived from natural biomolecules including chitosan, cellulose, albumin, gelatin, and lipids) [6]. Another category known as “green-synthesized nanoparticles” are typically inorganic nanoparticles produced using biological reducing and stabilizing agents such as plant extracts or microbial metabolites. In this review, the term “bio-based nanomaterials” is adopted as a broad classification framework for naturally derived nanocarrier systems used in drug delivery [7]. Conventional drug delivery systems are frequently limited by poor aqueous solubility, rapid metabolic degradation, low bioavailability, nonspecific distribution, and inadequate penetration across biological barriers. These limitations are particularly evident for many natural bioactive compounds, including polyphenols, alkaloids, and terpenoids, whose therapeutic potential is often restricted by unfavorable physicochemical properties [8]. Nanotechnology-based drug delivery systems address these challenges by improving drug stability, solubility, circulation time, intracellular uptake, and controlled release behavior [9,10]. Nanoscale carriers also enable passive targeting through the enhanced permeability and retention (EPR) effect, active targeting via ligand–receptor interactions, and stimuli-responsive drug release mechanisms that improve therapeutic precision and reduce systemic toxicity [11]. Bio-based nanocarriers can be broadly classified into polysaccharide-based, protein-based, lipid-based, inorganic green-synthesized, and hybrid nanomaterials with each class exhibits distinct advantages and limitations depending on composition, stability, drug loading capacity, release kinetics, and translational potential [12]. Lipid-based nanoparticles, for example, demonstrate high encapsulation efficiency and clinical success in nucleic acid delivery, whereas polysaccharide- and protein-based systems offer strong mucoadhesive, biodegradable, and receptor-mediated transport properties [13,14]. Green-synthesized metallic nanoparticles provide additional optical and theranostic functionalities but may present concerns related to long-term biodistribution and toxicity [15] (see Fig. 1).

Nevertheless, despite major advancements in nanomedicine, significant translational challenges [16] including large-scale manufacturing, batch reproducibility, long-term safety evaluation, regulatory approval, and inconsistent classification frameworks remain [[17], [18], [19]]. Current literature also lacks standardized benchmarking systems for comparing nanocarrier performance across different material platforms. Hence, this review provides an updated overview of bio-based nanomaterials in drug delivery, focusing on their classification, mechanisms, biomedical applications, translational challenges, and future research directions. Emphasis is placed on comparative analysis, clinical relevance, and the need for standardized evaluation strategies to support the successful translation of natural nanomaterials into pharmaceutical applications.

## 2 Rationale for natural nanomaterials in drug delivery

Conventional drug therapies are often limited in their therapeutic effectiveness due to several intrinsic challenges associated with the physicochemical and pharmacokinetic properties of many pharmaceutical compounds [[20], [21], [22]]. One of the primary obstacles is the poor aqueous solubility of several drugs, particularly natural bioactive molecules such as polyphenols, alkaloids, and terpenoids [23]. Poor solubility directly affects drug absorption in the gastrointestinal tract, reducing bioavailability and consequently, therapeutic efficacy [24]. In addition to solubility constraints, many drugs are prone to rapid degradation in biological fluids caused by enzymatic activity, acidic pH, or oxidative stress, which further diminishes their effective concentration in systemic circulation [25]. These limitations are compounded by the inability of conventional formulations to efficiently penetrate physiological barriers, such as cellular membranes, the blood–brain barrier, or mucosal surfaces, which restricts the drug's access to target tissues and organs [26]. Different classes of nanocarriers exhibit distinct functional advantages and limitations depending on their material composition. Polysaccharide-based systems such as chitosan and alginate are widely reported to provide excellent biocompatibility and mucoadhesion, although their drug loading capacity may be moderate depending on formulation conditions. Lipid-based nanocarriers, including liposomes and solid lipid nanoparticles, demonstrate high membrane compatibility and encapsulation efficiency, but may suffer from physical instability and leakage during storage. In contrast, inorganic or green-synthesized metallic nanoparticles such as gold and silver nanoparticles exhibit strong optical and therapeutic functionalities, including imaging and photothermal effects. However, concerns remain regarding long-term biodistribution, persistence, and potential toxicity in biological systems [27]. Natural nanomaterials provide an innovative solution to these challenges by serving as nanoscale carrier platforms that significantly enhance the stability of therapeutic agents. The nanoscale size allows for increased surface area and greater interaction with biological membranes, facilitating improved cellular uptake and penetration of target tissues [28]. Encapsulation of drugs within natural nanomaterials protects them from premature degradation and enzymatic attack in systemic circulation, prolonging the half-life of active molecules and ensuring that a higher fraction of the administered dose reaches the intended site of action. Furthermore, these nanoscale carriers enable controlled and sustained drug release, which maintains therapeutic levels of drugs over extended periods and reduces the frequency of dosing, improving patient compliance [15]. Additionally, natural nanomaterials can reduce systemic toxicity by concentrating drug delivery to specific tissues or pathological sites, such as tumors or inflamed regions, thereby minimizing exposure to healthy tissues [27]. This targeted delivery is achieved through both passive mechanisms, such as the enhanced permeability and retention (EPR) effect, and active mechanisms, such as surface functionalization with ligands, antibodies, or peptides that recognize specific cellular receptors. The ability to direct therapeutic agents selectively improves efficacy and enhances safety, particularly for drugs with narrow therapeutic windows [29]. Another key advantage of natural nanomaterials lies in their intrinsic biological compatibility. Derived from naturally occurring biomolecules, these nanomaterials exhibit low toxicity and reduced immunogenicity, which is critical for clinical translation. For example, polysaccharides such as chitosan, alginate, and cellulose, or protein-based carriers such as albumin and gelatin, are recognized as biologically benign, reducing the risk of adverse immune responses [30]. Their biodegradability ensures breakdown into non-toxic byproducts, minimizing long-term accumulation and chronic toxicity risks [31]. The versatility of natural nanomaterials further enhances their therapeutic potential. Surface functionalization with targeting moieties, such as folate, transferrin, peptides, or antibodies, enables precision drug delivery systems that actively recognize specific cells or tissues [32,33]. In addition to their functional advantages, the eco-friendly synthesis of natural nanomaterials supports sustainable nanotechnology approaches. Biomolecules such as plant extracts, microbial metabolites, polysaccharides, and proteins act as reducing and stabilizing agents during nanoparticle synthesis, eliminating the need for harsh chemicals or high-energy processes. This green synthesis approach enhances biocompatibility and reduces environmental impact [21]. Collectively, the rationale for employing natural nanomaterials in drug delivery is rooted in their ability to enhance therapeutic efficacy, minimize toxicity, and improve patient compliance. These systems address multiple limitations of conventional formulations by providing protective encapsulation, targeted delivery, controlled release, and biologically compatible platforms for both small-molecule and macromolecular therapeutics [34]. However, despite these advancements, several scientific hypotheses remain under active investigation, including the mechanistic role of nanoparticle surface chemistry in cellular uptake, the influence of protein corona formation on biological identity, and variability of the enhanced permeability and retention (EPR) effect across tumor. Unresolved issues also persist regarding long-term biodistribution, nano–bio interactions, and unpredictable in vivo behavior of multifunctional nanocarriers under physiological conditions, indicating that current understanding remains largely model-based and requires further experimental validation [35]. Furthermore, reproducibility of green synthesis methods and scalability of naturally derived nanocarriers remain major concerns, representing a critical translational bottleneck in clinical development. The authors highlight that the major bottleneck in nanomedicine is not material diversity but the absence of standardized benchmarking systems for comparing drug loading, release kinetics, and in vivo performance across different nanocarrier platforms [36,37].

## 3 Classification of natural nanomaterials in drug delivery

Recent developments in nanomedicine accentuate that classification of drug delivery nanocarriers should be based on chemical composition and material identity rather than synthesis route or size-based grouping, to improve consistency, regulatory alignment, and comparative evaluation of performance metrics such as drug loading, release kinetics, and biocompatibility [38]. Accordingly, natural nanomaterials can be systematically categorized into polysaccharide-based, protein-based, lipid-based, inorganic (including green-synthesized), and hybrid nanomaterials.

### 3.1 Polysaccharide-based nanomaterials

Polysaccharide-based nanomaterials are derived from naturally occurring biopolymers such as chitosan, alginate, cellulose, starch, and dextran and vary in size between 1 and 1000 nm. These materials are widely recognized for their biodegradability, mucoadhesion, and low immunogenicity, making them suitable for oral, nasal, and gastrointestinal drug delivery applications [39]. According to recent reviews, polysaccharides provide versatile chemical functionality that allows controlled drug encapsulation and site-specific release, particularly in pH-responsive environments. For example, chitosan-based nanoparticles (bromelain enzyme) exhibit high encapsulation efficiency greater than 80% and are suitable for enhancing mucosal adhesion and improving intestinal absorption of poorly soluble drugs, while alginate systems (lippie sidoides essential oil) with an encapsulation efficiency less than chitosan (55%) form hydrogel networks that enable sustained and controlled release [40]. Moreover, hydrophilic polysaccharides including hyaluronic acid and dextran decrease protein opsonization and uptake by the mononuclear phagoytic system, this consequently increases drug circulation half-life. In comparison with lipid-based nanoparticles, polysaccharide systems have better modularity and immunogenicity. Despite these advantages, polysaccharide-based systems often suffer from batch-to-batch variability, moisture sensitivity, and moderate mechanical strength, which limit industrial scalability and long-term formulation stability. Their clinical translation is also limited by pH-dependent instability.

### 3.2 Protein-based nanomaterials

Protein-based nanocarriers include self-assembling protein nanocages, albumin, gelatin, silk fibroin, casein, and ferritin. These systems are increasingly studied due to their intrinsic biological compatibility and ability to interact with endogenous transport pathways [41]. Due to their defined internal cavity, encapsulation efficiency usually depends on the loading method employed. Albumin nanoparticles (70-90% encapsulation efficiency) in particular have been extensively investigated in drug delivery due to their natural role as plasma transport proteins and ability to exhibit high loading for hydrophobic drugs through hydrophobic pockets. The protein shells of these nanocarriers protect siRNA and mRNA from degradation by RNase. Moreover, their surfaces are easily modifiable with ligands, affibodies and antibodies for receptor-mediated uptake making them highly effective for systemic and intestinal delivery applications [42]. Protein-based nanomaterials optimize siRNA delivery also support controlled enzymatic degradation, allowing predictable drug release profiles. Encapsulin, ferritin and lumazine synthase are often employed as scaffolds for gene and mRNA delivery to protect cargo and enable multivalent targeting. Compared to polysaccharide-based nanomaterials, protein-based nanomaterials have moderate encapsulation efficiency, are better options for vaccines and strong antigen presentation and have easier chemical/genetic modification possibilities. However, challenges such as protein denaturation, structural instability under processing conditions, and potential immunogenicity remain key limitations. Moreover, proteins can trigger immune and anti-drug antibodies by macrophages, leading to off-target release and granuloma formation.

### 3.3 Lipid-based nanomaterials

In contrast to metallic and polymeric nanocarriers, lipid-based nanomaterials including, liposomes, solid lipid nanoparticles (SLNs), nanostructured lipid carriers (NLCs), and lipid micelles are among the most clinically advanced nanocarriers due to their biomimetic structure similar to biological membranes [28]. They are particularly effective in encapsulating both hydrophilic and hydrophobic drugs and are widely used in RNA delivery and vaccine formulations. With encapsulation efficiency of 85-98%, lipid-based nanomaterials are clinically validated for mRNA. These protect mRNA release into the cytoplasm for protein synthesis. Moreover, their surfaces can be decorated with ligands for targeting and their cholesterol content contributes to structural stability and extended half-life by mitigating surface protein interactions [28]. Compared to polysaccharide- and protein-based nanomaterials, lipid-based nanomaterials are clinically proven for drug delivery and clinical translation. Their capacity to encapsulate both hydrophobic and hydrophilic compounds has contributed to their extensive use in anticancer and nucleic acid drug delivery. However, limitations such as drug leakage, oxidative instability, and relatively short shelf life remain major formulation challenges.

### 3.4 Inorganic (green-synthesized) nanomaterials

Inorganic nanomaterials include metallic nanoparticles such as gold, silver, iron oxide, mesoporous silica and zinc oxide. When synthesized using plant extracts, microbial metabolites, or enzymes, they are referred to as green-synthesized nanoparticles [43]. A widely accepted classification principle in nanomedicine literature is that these materials should be categorized based on their metallic core composition rather than synthesis method, as green synthesis only modifies the production route and not the material identity. Due to their surface area and porous structures, these nanoparticles have high drug loading capacities. Encapsulation efficiencies range from 60 to 90% for small molecules. Although promising, inorganic nanaomaterials are still in preclinical stage. Nevertheless, these materials exhibit unique optical, magnetic, and catalytic properties, enabling applications in drug delivery, imaging, photothermal therapy, and theranostics. Their surfaces are easy to modify and have excellent stability properties in addition to improved circulation. However, despite improved eco-friendly synthesis methods, concerns remain regarding long-term biodistribution, organ accumulation, and oxidative stress-induced toxicity, which continue to limit full clinical translation [43].

### 3.5 Hybrid nanomaterials

Hybrid nanomaterials integrate two or more material classes, typically combining organic (lipids, polymers, proteins) with inorganic nanoparticles to achieve multifunctional therapeutic performance. Recent literature highlights that hybrid systems are increasingly used in theranostic and precision medicine applications, where they combine drug delivery with imaging and targeting functions. They facilitate membrane fusion, prolong circulation and enable ligand conjugation. Lipid–polymer hybrid nanoparticles, for example, demonstrate improved circulation stability, controlled release, and enhanced tumor targeting efficiency [44]. However, their clinical translation is limited by complex synthesis procedures, scalability challenges, regulatory uncertainty, and batch-to-batch variability, making standardization a major research priority. Table 1 below shows the classification of natural nanomaterials in drug delivery, Table 2 also gives the comparative performance of natural nanomaterials in drug delivery.

**Table 1:** Classification of natural nanomaterials in drug delivery.

Type of Nanomaterial	Source/Origin	Advantages in Drug Delivery	Examples/Applications
Green-Synthesized Metallic Nanoparticles	Plant extracts, microbial metabolites	Biocompatible, stable, high surface area, multifunctional, allows drug loading and imaging	Gold nanoparticles for anticancer drugs; silver nanoparticles for antimicrobial delivery [45].
Polysaccharide and Biopolymeric Nanomaterials	Chitosan, cellulose, alginate, gelatin	Biodegradable, biocompatible, mucoadhesive, surface functionalization for targeting, sustained/controlled release	Chitosan nanoparticles for oral/ocular delivery; alginate hydrogels for protein and vaccine delivery [46].
Natural Small Molecule-Based Nanomaterials	Triterpenoids, sterols, alkaloids, polysaccharides	Self-assembly, amphiphilic behavior, stimuli-responsive, improved solubility and bioavailability	Saponin micelles for hydrophobic drugs; alkaloid nanoparticles for targeted cancer therapy [47].
Hybrid/Exosome-Mimetic Nanomaterials	Combination of natural polymers + synthetic materials, cell membranes	Enhanced stability, immune evasion, multifunctional, targeted delivery	Exosome-coated nanoparticles for gene/drug delivery; polymer-coated gold nanoparticles for imaging therapy [48].

**Table 2:** Comparative performance of natural nanomaterials in drug delivery.

Nanomaterial Type	Drug Loading Capacity	Release Mechanism	Scalability	Stability	Clinical/Regulatory Status	Toxicity	Encapsulation efficiency	Key Limitations
Polysaccharide-based (chitosan, alginate, cellulose)	Reported as effective carriers with moderate to high encapsulation depending on formulation method	pH-responsive, enzymatic degradation, and mucoadhesive controlled release widely reported	Generally scalable due to abundant natural sources, but extraction variability affects reproducibility	Good stability in solid form; sensitive to moisture and ionic conditions	Widely investigated in preclinical drug delivery systems	Very low	40-90%	Batch variability, purification differences, moisture sensitivity
Protein-based (albumin, gelatin, silk fibroin)	High affinity binding for hydrophobic drugs; albumin nanoparticles widely used for drug encapsulation	Enzymatic degradation and receptor-mediated release mechanisms	Moderately scalable; albumin systems already industrially used in pharmaceuticals	Moderate stability; proteins may denature under heat/pH changes	Some systems (e.g., albumin-based nanoparticles) are clinically established	Low	40-80%	Structural instability, denaturation risk, storage limitations
Lipid-based (liposomes, SLN, NLC)	High encapsulation efficiency especially for lipophilic drugs and nucleic acids	Controlled release *via* lipid bilayer diffusion and membrane fusion	High scalability; lipid nanoparticles are industrially established	Moderate stability; leakage and oxidation are known issues	Most clinically advanced nanocarrier system (FDA-approved lipid formulations exist)	Low	85-98%	Drug leakage, short shelf life, oxidative instability
Inorganic green-synthesized nanoparticles (Ag, Au, Fe_3_O_4_)	High surface adsorption capacity due to large surface area	Limited intrinsic release control; mostly surface functionalization-based	Low to moderate due to synthesis variability	High physical stability but prone to aggregation without coating	Mostly preclinical; limited clinical translation	Low tolerability risk	50-80%	Long-term toxicity, accumulation, unclear biodistribution

Hybrid nanomaterials (polymer–lipid, polymer–metal, exosome-mimetic systems)	Very high due to multi-compartment encapsulation	Multi-stimuli responsive controlled release	Moderate to low due to complex synthesis	High structural stability but dependent on design	Emerging translational research	Lower than pure cationic polymers	80-95%	Complex fabrication, regulatory uncertainty

The comparative analysis of natural nanomaterials presented in Table 2 summarizes key reported trends in drug loading capacity, release mechanisms, scalability, stability, and translational status across major nanocarrier systems, including polysaccharide-based, protein-based, lipid-based, inorganic green-synthesized, self-assembled small molecule, and hybrid nanomaterials, as reported in recent nanomedicine studies and review articles. Despite the broad advantages of natural nanomaterials, a critical comparison indicates that no single system fully satisfies all requirements for drug delivery. Moreover, the integration of multiple material properties within advanced nanocarrier systems is therefore an area of ongoing research aimed at overcoming individual limitations and improving overall delivery performance [[49], [50], [51]].

The diagram illustrates the main types of natural nanomaterials employed in drug delivery, including green-synthesized metallic nanoparticles, polysaccharide and biopolymeric nanomaterials, natural small molecule-based nanomaterials, and hybrid/exosome-mimetic nanomaterials. It shows their biological sources, key advantages such as biocompatibility, biodegradability, and controlled release, and representative examples of applications in drug delivery, highlighting their versatility and therapeutic potential.

Overall, adopting a unified material-based classification system improves the clarity and consistency of evaluating nanocarrier performance in drug delivery research. Such an approach allows better comparison of key parameters including delivery efficiency, biological interaction, and therapeutic performance across different nanomaterial platforms. Recent studies emphasize that material composition plays a central role in determining nanocarrier behavior, influencing factors such as stability, transport efficiency, and interaction with biological systems [52]. In addition, advances in clinically relevant systems such as lipid nanoparticles demonstrate how well-defined material frameworks contribute to improved delivery outcomes, particularly in nucleic acid therapeutics where optimized design enhances stability and intracellular delivery [53]. Similarly, protein-based systems such as albumin nanoparticles highlight the importance of material selection in achieving efficient drug transport and targeted delivery. Therefore, improving classification consistency and material-based evaluation is essential for enhancing comparability across studies and supporting the continued development and clinical translation of nanocarrier systems.

## 4 Mechanisms of Drug Delivery using natural nanomaterials

Natural nanomaterials enhance drug delivery through several complementary mechanisms that collectively improve the therapeutic efficacy of drugs while minimizing systemic toxicity. These mechanisms can be broadly categorized into passive targeting, active targeting, and stimuli-responsive controlled release, each of which utilizes distinct biological principles and nanomaterial properties to achieve effective drug localization and release [54]. Each mechanism addresses a particular limitation of conventional drug formulations such as poor solubility, low tissue specificity, rapid clearance, and systemic toxicity while their combination allows the development of multifunctional nanocarriers. Understanding and optimizing these mechanisms is essential for the translation of natural nanomaterials into clinically effective therapies for cancer, infectious diseases, neurological disorders, and other challenging conditions [25].

### 4.1 Passive targeting

Passive targeting relies primarily on the enhanced permeability and retention (EPR) effect, a phenomenon observed in many pathological tissues, including solid tumors, inflamed regions, and ischemic sites [55]. Nanoparticles within the size range of 50–200 nm can accumulate preferentially in these tissues due to their leaky vasculature and impaired lymphatic drainage. Unlike conventional drugs that diffuse randomly through tissues and blood circulation, nanoscale carriers exploit these structural abnormalities to concentrate therapeutic agents where they are most needed, without requiring any specific ligand–receptor interactions [56]. Natural nanomaterials, including polysaccharide nanoparticles and green-synthesized metallic nanoparticles, are particularly well-suited for passive targeting because their biocompatibility and surface properties facilitate extended circulation times in the bloodstream. Extended circulation enhances the likelihood that nanoparticles will encounter pathological tissues exhibiting the EPR effect, thereby improving drug accumulation at target sites [57]. Additionally, the nanoscale size prevents rapid renal clearance, while surface modifications with hydrophilic polymers, such as polyethylene glycol (PEG), can further prolong systemic residence and reduce opsonization by the immune system. Passive targeting thus provides a straightforward yet powerful mechanism for increasing drug concentrations at disease sites while limiting exposure to healthy tissues [58].

### 4.2 Active targeting

Active targeting involves the functionalization of nanocarriers with specific ligands, peptides, antibodies, or small molecules that selectively bind to cell surface receptors or biomarkers expressed predominantly on diseased cells. This mechanism allows nanomaterials to recognize and adhere to target tissues with high specificity, enhancing therapeutic precision and minimizing off-target effects. Examples include folate-functionalized nanoparticles targeting folate receptors overexpressed in certain cancer cells, transferrin-conjugated nanocarriers for targeted brain delivery, and antibody-functionalized carriers for immune-targeted therapies [59]. Natural nanomaterials offer an advantage in active targeting because their surfaces can be readily modified using chemical or enzymatic conjugation methods without compromising biocompatibility. For instance, chitosan and alginate nanoparticles provide functional groups that can attach targeting ligands, while lipid-based nanocarriers such as nanoliposomes can incorporate ligands directly into the lipid bilayer [60]. Active targeting not only enhances drug accumulation in diseased tissues but also facilitates cellular uptake through receptor-mediated endocytosis, improving intracellular delivery of therapeutic agents, including small-molecule drugs, peptides, and nucleic acids. This precise delivery reduces the required therapeutic dose, mitigates systemic side effects, and can enhance the efficacy of otherwise poorly bioavailable drugs [61].

### 4.3 Stimuli-responsive controlled release

Stimuli-responsive drug release leverages physiological or pathological cues to trigger the release of encapsulated drugs at the site of interest [62]. Natural nanomaterials can be engineered to respond to various internal stimuli, including pH variations, redox conditions, enzymatic activity, or temperature differences, which are often characteristic of diseased tissues. For example, tumor microenvironments typically exhibit slightly acidic pH, elevated levels of reactive oxygen species (ROS), and overexpressed enzymes such as matrix metalloproteinases (MMPs). Nanocarriers sensitive to these triggers release drugs preferentially within tumors, maximizing efficacy while limiting systemic exposure [63]. Polysaccharide-based nanomaterials, such as chitosan and alginate nanoparticles, can be designed to degrade under acidic conditions or enzymatic cleavage, enabling site-specific release. Similarly, hybrid nanocarriers incorporating metallic nanoparticles or small-molecule natural compounds can respond to external stimuli, such as heat or magnetic fields, to induce drug release [64]. Stimuli-responsive delivery not only increases therapeutic precision but also allows controlled, sustained, or pulsatile drug release, which is particularly advantageous for drugs with short half-lives or those requiring precise temporal dosing. This smart drug release system represents one of the most sophisticated mechanisms by which natural nanomaterials improve drug delivery outcomes [65].

### 4.4 Integration of multiple mechanisms

Many natural nanomaterials are engineered to combine these three mechanisms passive targeting, active targeting, and stimuli-responsive release to achieve multifunctional drug delivery systems. For instance, a chitosan-coated gold nanoparticle may exploit passive targeting *via* the EPR effect, actively target cancer cells through folate ligands, and release drugs in response to acidic tumor microenvironments [66]. This integration maximizes therapeutic efficiency while minimizing side effects, representing the cutting-edge approach in nanomedicine. By leveraging the inherent biocompatibility, tunable surface chemistry, and structural versatility of natural nanomaterials, such systems provide precision therapy, enhanced bioavailability, and prolonged circulation time simultaneously [67].

The figure depicts the primary mechanisms by which natural nanomaterials enhance drug delivery. Passive targeting leverages the enhanced permeability and retention (EPR) effect for accumulation in tissues with leaky vasculature. Active targeting involves surface functionalization of nanocarriers with ligands or antibodies that recognize specific cellular receptors, enhancing site-specific delivery. Stimuli-responsive controlled release enables drug liberation in response to internal cues (e.g., pH, enzymes) or external stimuli (e.g., temperature), improving therapeutic precision and minimizing systemic side effects. Examples of nanomaterials, such as chitosan nanoparticles, gold nanoparticles, and nanoliposomes, are shown in the context of these delivery mechanisms. Recent advances in nanomedicine consistently demonstrate that the therapeutic effectiveness of natural nanomaterials is governed by the integration of three core mechanisms: passive targeting, active targeting, and stimuli-responsive controlled release**.** These mechanisms do not function independently; rather, they operate synergistically to improve drug accumulation, cellular uptake, and site-specific release across different biomedical applications, particularly in oncology, gastrointestinal delivery, neurological disorders, and gene therapy systems discussed in the following section [68].

## 5 Applications of natural nanomaterials in drug delivery

The applications of natural nanomaterials in drug delivery are closely linked to the underlying design and functional behavior of nanocarrier systems. These systems are developed to improve delivery efficiency, enhance interaction with biological environments, and enable controlled transport of therapeutic agents. Advances in nanocarrier platforms, particularly lipid-based systems, demonstrate how optimized design contributes to improved intracellular delivery and therapeutic performance, especially in nucleic acid delivery applications [69]. In addition, biomaterial-based nanostructures support effective interaction with biological systems, facilitating improved delivery and functional integration within tissues. Protein-based systems such as albumin nanoparticles further enhance drug transport and distribution, contributing to improved targeting efficiency and therapeutic outcomes in systemic applications. Understanding how these design principles influence delivery performance is essential for evaluating the effectiveness and translational potential of nanomaterial-based drug delivery systems across different biomedical applications [70,71]. By enhancing drug solubility, stability, and bioavailability, these nanomaterials can improve therapeutic outcomes while reducing adverse side effects commonly associated with conventional drug formulations [72].

### 5.1 Cancer therapy

Cancer remains one of the most extensively investigated applications of bio-based nanomaterial-mediated drug delivery. Natural nanocarriers such as chitosan, alginate, and green-synthesized gold nanoparticles have demonstrated significant potential to improve the delivery of chemotherapeutic agents by enhancing drug solubility, protecting unstable compounds from degradation, and enabling controlled release within tumor microenvironments [73,74]. Through previously described passive and ligand-mediated active targeting mechanisms, these systems promote preferential drug accumulation in tumor tissues and improve intracellular uptake, thereby enhancing therapeutic efficacy while reducing systemic toxicity. In addition, stimuli-responsive nanocarriers capable of releasing drugs in response to tumor-specific conditions, including acidic pH, elevated glutathione levels, enzymatic activity, and hypoxia, have further improved targeting precision and localized therapeutic action. Compared with conventional chemotherapy, such multifunctional nanocarrier systems offer superior control over drug distribution and reduced off-target effects. However, the clinical effectiveness of passive targeting remains limited by tumor heterogeneity, inconsistent vascular permeability, and variable receptor expression, which may reduce delivery efficiency across different cancer types. Addressing these biological and translational barriers remains essential for the broader clinical application of natural nanomaterial-based cancer therapeutics [75,76].

### 5.2 Gastrointestinal disorders

Bio-based nanomaterials have demonstrated considerable potential in the treatment of gastrointestinal (GI) disorders, including inflammatory bowel disease, gastric ulcers, and intestinal infections. Oral drug delivery is frequently limited by poor absorption, enzymatic degradation, and instability within the acidic gastric environment [77]. Polysaccharide-based nanocarriers such as chitosan and alginate improve oral delivery by providing protective encapsulation, prolonged intestinal residence, and controlled drug release, thereby enhancing drug absorption and therapeutic efficacy [78]. Stimuli-responsive delivery systems play a particularly important role in GI applications by enabling site-specific release in response to physiological triggers such as pH variation and enzymatic activity along the gastrointestinal tract [79]. Due to their pH-sensitive and mucoadhesive properties, chitosan- and alginate-based nanoparticles exhibit enhanced intestinal retention and improved bioavailability of poorly soluble compounds while protecting encapsulated drugs from gastric degradation [80]. Compared with conventional oral formulations, these systems provide greater control over localized drug release and reduced systemic side effects. Nevertheless, variability in gastrointestinal transit time, mucus turnover, and local pH conditions may affect reproducibility of drug release and absorption, thereby limiting consistent clinical performance.

### 5.3 Delivery of natural bioactive agents

Several natural bioactive compounds, including polyphenols, flavonoids, alkaloids, and terpenoids, posses significant pharmacological potential but exhibit poor aqueous solubility, low stability, and rapid metabolic degradation, which restrict their clinical application [81]. Bio-based nanocarriers address these limitations by enhancing solubility, protecting encapsulated compounds from premature degradation, and enabling controlled and site-specific drug release. Preclinical studies involving curcumin-loaded chitosan nanoparticles and quercetin-loaded nanoliposomes have demonstrated improved bioavailability, enhanced intracellular uptake, and superior therapeutic activity compared with free compounds [82]. Lipid-, protein-, and polysaccharide-based nanocarriers each contribute distinct advantages in phytochemical delivery. Lipid-based systems improve membrane permeability and intracellular transport, whereas protein-based carriers such as albumin nanoparticles enhance systemic circulation and biological compatibility. Polysaccharide-based nanostructures additionally provide mucoadhesive and controlled-release properties that support prolonged drug retention. Through previously described passive targeting, ligand-mediated uptake, and stimuli-responsive release mechanisms, these systems improve tissue accumulation and therapeutic precision [83]. However, despite promising preclinical outcomes, the clinical translation of phytochemical-loaded nanocarriers remains limited by formulation instability, low large-scale reproducibility, and insufficient pharmacokinetic and long-term safety data.

### 5.4 Vaccines, proteins, and gene therapy

**Fig. 2:**
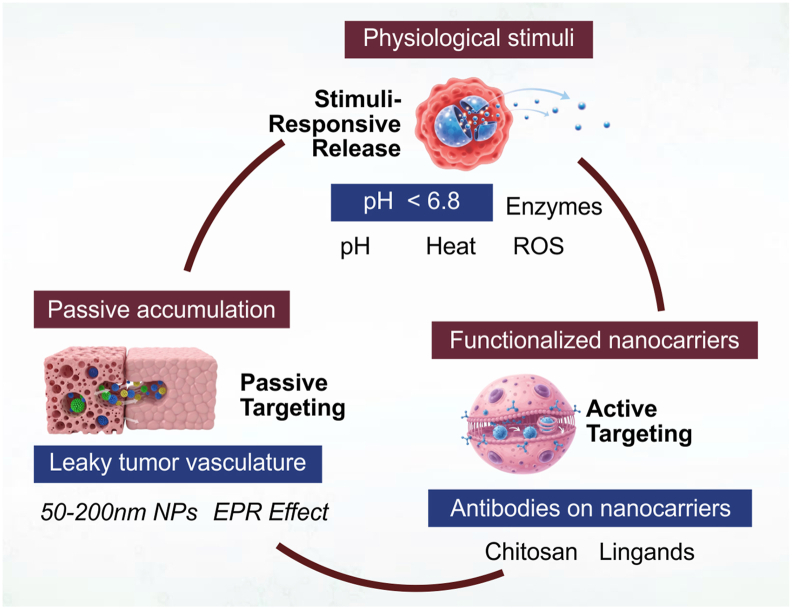
Mechanisms of Drug Delivery using Natural Nanomaterials: Passive, Active, and Stimuli-Responsive Pathways (This figure was produced using professional graphic software (CorelDRAW, Adobe Illustrator).

Bio-based nanomaterials have become increasingly important in the delivery of macromolecular therapeutics, including vaccines, proteins, and nucleic acids, whose conventional administration is often limited by enzymatic degradation, poor cellular uptake, and low biological stability [84]. Biopolymeric nanocarriers such as chitosan, alginate, and protein-based nanoparticles enhance the stability of these fragile biomolecules through protective encapsulation and controlled intracellular delivery. Functionalization with targeting ligands or cell-penetrating peptides further improves tissue specificity and cellular uptake, thereby enhancing immunogenicity in vaccine applications and transfection efficiency in gene therapy [85]. Among available systems, lipid-based and hybrid nanocarriers currently demonstrate the greatest clinical translation potential for nucleic acid delivery due to their high encapsulation efficiency, favorable biocompatibility, and ability to facilitate endosomal escape [86,87]. These properties have contributed significantly to the success of mRNA vaccines and emerging RNA- and CRISPR-based therapeutics. In addition, stimuli-responsive systems capable of releasing therapeutic cargo in response to endosomal pH or redox conditions improve intracellular delivery precision and functional gene expression. Nevertheless, challenges including immune activation, off-target effects, storage instability, and large-scale manufacturing complexity continue to limit the widespread clinical application of many macromolecular nanocarrier systems (see Fig. 2).

### 5.5 Neurological and other disorders

**Fig. 3:**
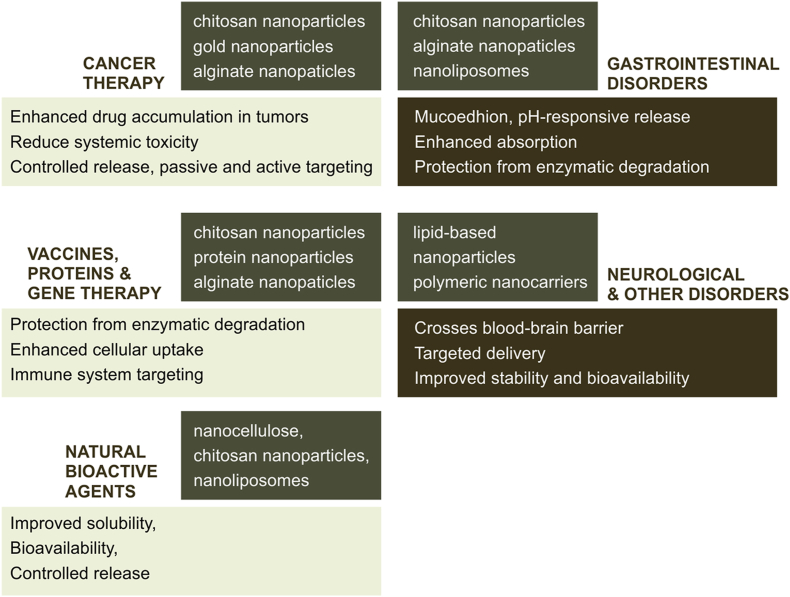
Applications of Natural Nanomaterials in Drug Delivery across Therapeutic Areas (This figure was produced using professional graphic software (CorelDRAW, Adobe Illustrator).

Natural nanomaterials also provide solutions for drug delivery across challenging biological barriers, such as the blood–brain barrier (BBB). Lipid-based nanoparticles, polymeric nanocarriers, and small molecule-derived nanostructures can be engineered to cross the BBB via receptor-mediated transcytosis or by exploiting nanoparticle size and surface properties [88]. This capability enables the delivery of drugs for neurodegenerative diseases, brain tumors, and central nervous system infections that are otherwise difficult to treat. Additionally, nanocarriers are applied in ophthalmology, pulmonary therapy, and cardiovascular drug delivery, demonstrating broad applicability across multiple therapeutic areas [89]. Neurological applications rely heavily on active targeting and size-dependent passive transport mechanisms, particularly for crossing the blood BBB. Nanocarriers functionalized with targeting ligands (e.g., transferrin, lactoferrin, or peptides) enhance receptor-mediated transcytosis across endothelial cells of the BBB [90]. Recent studies confirm that active targeting significantly improves brain uptake and therapeutic delivery of neuroprotective agents and gene-based therapeutics compared to non-modified systems. In addition, nanoscale size (typically <100 nm) enables limited passive diffusion across disrupted BBB regions in pathological conditions such as Alzheimer's disease and brain tumors. Thus, neurological delivery systems depend on a combined passive–active targeting strategy, with stimuli-responsive systems further enhancing intracellular release in diseased brain tissues [91]. Table 3 and Fig. 3 below give summaries of the applications of natural nanomaterials in drug delivery. This capability enables the delivery of drugs for neurodegenerative diseases, brain tumors, and central nervous system infections that are otherwise difficult to treat. Additionally, nanocarriers are applied in ophthalmology, pulmonary therapy, and cardiovascular drug delivery, demonstrating broad applicability across multiple therapeutic areas.

**Table 3:** Applications of natural nanomaterials in drug delivery.

Therapeutic Area	Type of Natural Nanomaterial	Advantages in Drug Delivery	Examples/References
Cancer Therapy	Chitosan nanoparticles, alginate nanoparticles, green-synthesized gold nanoparticles	Enhanced drug accumulation in tumors, reduced systemic toxicity, controlled release, passive and active targeting	Gold nanoparticles delivering doxorubicin; chitosan nanoparticles for anticancer drugs [92].
Gastrointestinal Disorders	Chitosan, alginate, nanoliposomes	Mucoadhesion, protection from enzymatic degradation, enhanced absorption, pH-responsive release	Chitosan-alginate nanoparticles for ulcerative colitis treatment [90].
Natural Bioactive Agents	Nanocellulose, chitosan nanoparticles, nanoliposomes	Improved solubility, stability, bioavailability, controlled release	Curcumin-loaded chitosan nanoparticles; quercetin nanoliposomes [93].
Vaccines, Proteins, and Gene Therapy	Chitosan, alginate, protein-based nanoparticles	Protection from enzymatic degradation, enhanced cellular uptake, immune system targeting, stimuli-responsive release	mRNA vaccine delivery via chitosan nanoparticles; protein therapeutics [94]
Neurological and Other Disorders	Lipid-based nanoparticles, polymeric nanocarriers, small molecule-based nanomaterials	Crosses blood–brain barrier, targeted delivery, improved stability and bioavailability	Delivery of drugs for brain tumors and neurodegenerative diseases [95].

The figure illustrates the diverse therapeutic applications of natural nanomaterials in drug delivery. The central node represents natural nanomaterials, which connect to five major therapeutic areas: cancer therapy, gastrointestinal disorders, delivery of natural bioactive agents, vaccines/proteins/gene therapy, and neurological or other disorders. For each area, the figure highlights the types of nanomaterials commonly used (such as chitosan nanoparticles, gold nanoparticles, alginate nanoparticles, and nanoliposomes), their key advantages including enhanced solubility, targeted delivery, controlled release, and protection from degradation, and representative examples of drug delivery applications. This visual summary demonstrates how natural nanomaterials serve as versatile platforms to improve drug bioavailability, efficacy, and safety across multiple medical contexts.

## 6 Challenges in clinical translation of natural nanomaterials

Despite substantial advancements in the design and application of natural nanomaterials for drug delivery, several critical challenges continue to hinder their successful translation from laboratory research to clinical use. These challenges span issues related to raw material variability, manufacturing reproducibility, regulatory approval, safety evaluation, and long-term in vivo performance [96].

### 6.1 Variability and reproducibility

Despite promising therapeutic applications, the clinical translation of bio-based nanomaterials remains significantly constrained by variability and reproducibility challenges. Because these materials are frequently derived from plant, microbial, or animal sources, their physicochemical composition may vary according to geographical origin, seasonal conditions, cultivation methods, and extraction procedures [97]. Such variability directly influences nanoparticle characteristics including particle size, surface charge, encapsulation efficiency, and release kinetics, thereby affecting therapeutic performance and safety profiles. Batch-to-batch inconsistency remains a major obstacle to large-scale manufacturing and regulatory approval, particularly for systems requiring precise formulation control and long-term stability. Compared with fully synthetic nanocarriers, naturally derived systems often exhibit greater compositional heterogeneity, complicating quality assurance and reproducible industrial production. Consequently, standardized protocols for raw material sourcing, extraction, purification, characterization, and formulation are essential to improve manufacturing consistency and support clinical translation [98]. Beyond manufacturing variability, the clinical translation of nanocarrier systems is influenced by multiple biological and translational challenges that affect delivery performance and therapeutic consistency. Recent advances in nanomedicine highlight that although lipid-based nanocarriers improve delivery efficiency and pharmacokinetic behavior, their performance can still vary depending on biological conditions and formulation characteristics. In addition, biomaterial-based systems demonstrate strong interaction with biological environments, but their behavior can differ across physiological conditions, influencing overall delivery outcomes and reproducibility [99]. Protein-based nanocarriers such as albumin nanoparticles also show variability in transport efficiency depending on formulation and biological context. These variations contribute to challenges in achieving consistent therapeutic performance, highlighting the importance of improving reproducibility and understanding nanocarrier–biological system interactions for successful clinical translation.

### 6.2 Regulatory and quality control challenges

The regulatory framework governing bio-based nanomaterial drug delivery systems remains insufficiently standardized and continues to evolve alongside advances in nanomedicine. Regulatory approval requires comprehensive evaluation of safety, efficacy, pharmacokinetics, biodistribution, and long-term toxicity prior to clinical application. However, compared with synthetic nanocarriers, naturally derived nanomaterials frequently exhibit greater compositional heterogeneity due to the presence of multiple bioactive constituents, thereby complicating quality control and physicochemical characterization [100]. Critical parameters including particle size distribution, surface chemistry, encapsulation efficiency, sterility, storage stability, and batch reproducibility must be rigorously validated to satisfy regulatory requirements. The absence of universally accepted characterization protocols and standardized evaluation frameworks for natural nanocarriers further contributes to prolonged approval timelines and increased development costs. In addition, differences in international regulatory policies may complicate global commercialization and clinical implementation. Early collaboration with regulatory agencies and the establishment of standardized manufacturing and characterization guidelines are therefore essential to facilitate successful clinical translation and regulatory acceptance of bio-based nanomedicine systems.

### 6.3 Safety and toxicity considerations

Although bio-based nanomaterials are generally regarded as biocompatible and biodegradable, comprehensive safety evaluation remains essential prior to clinical application. Potential concerns include immune activation, unintended off-target accumulation, oxidative stress, and prolonged retention within major organs such as the liver, spleen, and kidneys [101]. Long-term exposure may result in cumulative toxicity or altered physiological responses, particularly for systems intended for chronic administration. Nanoparticle interactions with biological fluids, plasma proteins, immune cells, and tissue microenvironments can further modify biodistribution, cellular uptake, clearance pathways, and therapeutic performance. Such interactions may lead to unpredictable pharmacokinetic behavior and variable toxicity profiles across different biological systems. Compared with conventional drug formulations, certain nanocarriers may also exhibit increased risk of immunogenicity or organ-specific accumulation due to their nanoscale properties. Consequently, systematic preclinical evaluation involving long-term biodistribution, pharmacokinetic profiling, immunogenicity assessment, and dose-dependent cytotoxicity studies across multiple animal models is required to establish the safety and clinical reliability of bio-based nanomaterial delivery systems [102].

### 6.4 Scalability and manufacturing challenges

Scaling up laboratory protocols for natural nanomaterial synthesis to industrial-scale production remains a significant hurdle. Processes that are feasible on a small scale, such as green synthesis using plant extracts or microbial biomolecules, may not be easily adaptable to large-scale production without compromising particle uniformity, reproducibility, or bioactivity [103]. Moreover, maintaining sterility, controlling particle size distribution, and ensuring batch-to-batch consistency require sophisticated manufacturing technologies and robust quality control measures. Investment in scalable, cost-effective, and standardized production techniques is therefore essential to facilitate the clinical translation of natural nanomaterials [104]. A major reason why many nanomaterial-based drug delivery systems remain at the preclinical stage is the gap between laboratory-scale optimization and requirements for clinical translation. Although experimental studies often report improved drug loading efficiency, stability, and delivery performance under controlled conditions, these outcomes are frequently difficult to reproduce at larger scale due to variability in raw materials, formulation parameters, and processing conditions. This limitation has been widely recognized in nanomedicine literature as a key barrier to industrial translation of nanocarrier systems [105]. In addition, regulatory approval requires extensive evaluation of safety, reproducibility, and long-term performance, which many nanomaterial platforms still struggle to fully demonstrate. Recent studies on clinically relevant systems such as lipid nanoparticles emphasize that even advanced platforms require careful optimization to maintain stability and functional performance during scale-up and storage conditions. Similarly, biomaterial-based systems, including protein and polymeric nanocarriers, show strong potential for biomedical applications but still face challenges related to consistency and manufacturing reproducibility. Overall, clinical translation is further complicated by complex biological interactions that influence nanocarrier performance in vivo, making it difficult to fully predict therapeutic outcomes based on preclinical models alone. As a result, only a limited number of nanocarrier systems particularly lipid-based and protein-based platforms have successfully progressed toward clinical application, highlighting the persistent gap between experimental innovation and real-world pharmaceutical implementation [105]. From a critical standpoint, current evidence suggests that translational failure is driven not only by engineering limitations but also by differences between simplified preclinical models and the complexity of human physiological environments, which remain difficult to fully replicate in laboratory studies.

### 6.5 Safety evaluation: toxicity, immunogenicity, and long-term effects

Safety evaluation is a fundamental requirement for the successful clinical translation of bio-based nanocarrier systems. Although biomaterial-, protein-, and lipid-based nanostructures are generally considered biocompatible due to their biological origin, their in vivo safety profiles remain highly dependent on formulation composition, particle size, surface chemistry, dosage, and route of administration [106,107]. Consequently, biocompatibility alone does not guarantee long-term clinical safety. Recent studies demonstrate that nanocarrier interactions with immune cells, plasma proteins, and biological membranes significantly influence biodistribution, clearance behavior, therapeutic efficacy, and toxicity. Immune activation, complement responses, off-target accumulation, and prolonged organ retention may occur depending on nanocarrier physicochemical properties and exposure duration. In addition, repeated or chronic administration may increase the risk of cumulative toxicity and immunogenicity, particularly in systemic delivery applications. Among current platforms, lipid-based nanocarriers have shown relatively strong clinical safety profiles, largely due to advances in formulation optimization and regulatory experience gained through mRNA vaccine development. However, protein- and polysaccharide-based systems still require extensive long-term evaluation to establish consistent pharmacokinetic behavior and immunological safety. Therefore, comprehensive assessment of toxicity, immunogenicity, biodistribution, and long-term biological effects remains essential for the development of clinically reliable nanomaterial-based drug delivery systems [106].

#### 6.5.1 Acute and chronic toxicity studies

Acute toxicity studies are essential for assessing the immediate biological effects of nanocarrier exposure, including organ toxicity, hematological alterations, inflammatory responses, and behavioral changes following single or short-term administration. Bio-based nanocarriers such as chitosan, alginate, and lipid-based nanoparticles generally demonstrate low acute toxicity and favorable biocompatibility in preclinical studies, with minimal adverse effects on liver and kidney function [107]. Chitosan-based systems, in particular, exhibit low cytotoxicity and limited histopathological alterations due to their biodegradable and non-toxic polymeric structure. Similarly, lipid nanoparticle platforms used in drug and RNA delivery typically show acceptable short-term safety profiles, although transient inflammatory responses may occur depending on formulation composition, particle size, surface charge, and administered dose [108]. In contrast, chronic toxicity studies evaluate the consequences of prolonged or repeated nanocarrier exposure and are therefore critical for predicting long-term clinical safety [109]. Although biodegradable polymeric systems generally degrade into relatively non-toxic byproducts, long-term biodistribution and clearance behavior remain strongly influenced by molecular weight, crosslinking density, surface modification, and physiological conditions [110,111]. Persistent accumulation of nanomaterials within organs such as the liver, spleen, and kidneys may increase the risk of oxidative stress, inflammatory responses, and organ-specific toxicity during extended administration. These concerns are particularly relevant for metallic nanoparticles, which may exhibit prolonged tissue retention even when synthesized through green or biologically mediated approaches. Consequently, comprehensive long-term evaluation involving biodistribution analysis, degradation profiling, immunotoxicity assessment, and chronic exposure studies is required to establish the clinical safety and regulatory reliability of bio-based nanocarrier systems.

#### 6.5.2 Immunogenicity

Immunogenicity remains an important safety consideration in the development of bio-based nanocarrier systems. Although natural nanomaterials generally exhibit lower immunogenicity than many synthetic nanoparticles, factors including particle size, surface charge, aggregation behavior, formulation impurities, and surface chemistry can still trigger innate or adaptive immune responses [112]. Residual biological contaminants or aggregated nanoparticles may activate macrophages, complement pathways, or inflammatory signaling mechanisms, potentially resulting in hypersensitivity reactions and reduced therapeutic safety [113].

Recent studies involving lipid nanoparticles for RNA delivery demonstrate that immune activation may still occur despite the favorable clinical biocompatibility of these systems, particularly when formulation composition or dosing conditions are not adequately optimized [114]. Similarly, albumin-based nanoparticles typically exhibit improved immune compatibility due to their endogenous protein origin; however, their immunological behavior may vary depending on disease state, administration route, and biological environment [115]. Biomaterial-based nanostructures used in regenerative medicine and therapeutic delivery also generally demonstrate favorable immune tolerance, although long-term immunological effects under repeated administration remain insufficiently characterized [116]. Consequently, minimizing immunogenicity requires careful optimization of nanoparticle purification, surface functionalization, size distribution, and formulation stability while preserving therapeutic performance. Comprehensive immunotoxicity assessment, including evaluation of cytokine response, complement activation, macrophage interaction, and repeated-dose immune effects, remains essential for the safe clinical translation of bio-based nanocarrier systems.

#### 6.5.3 Long-term safety and biodegradation

The long-term safety of bio-based nanocarriers is closely associated with their biodegradation behavior, clearance mechanisms, and persistence within biological systems. Natural polymers such as chitosan, alginate, and cellulose derivatives generally undergo enzymatic or hydrolytic degradation into biologically compatible byproducts, thereby reducing the likelihood of prolonged tissue accumulation and chronic toxicity [117]. Similarly, appropriately surface-modified green-synthesized metallic nanoparticles may exhibit improved biocompatibility and reduced oxidative stress, supporting more efficient systemic clearance. Recent evidence from lipid nanoparticle systems demonstrates that biodegradation and long-term safety are strongly influenced by formulation composition, particularly the use of ionizable and endogenous lipid-like materials that facilitate metabolic breakdown and post-delivery elimination [118,119]. Protein-based nanocarriers such as albumin nanoparticles also demonstrate favorable long-term safety profiles because enzymatic degradation produces naturally metabolizable amino acid components, thereby minimizing accumulation within major organs. In regenerative and therapeutic applications, biomaterial-based systems generally exhibit good tissue compatibility and controlled biodegradation behavior, although comprehensive long-term in vivo validation remains limited. Importantly, biodegradation kinetics should align with the therapeutic release profile of the encapsulated drug to ensure effective delivery while preventing residual nanoparticle retention. However, nanocarrier interactions with complex physiological environments remain highly dynamic and formulation-dependent, potentially influencing stability, clearance pathways, biodistribution, and therapeutic consistency [120]. Consequently, extended evaluation involving long-term biodistribution analysis, organ accumulation studies, blood biochemistry, histopathological assessment, and physiologically relevant in vivo models remains essential for accurately determining the long-term safety and clinical reliability of bio-based nanocarrier systems [121].

## 7 Case studies and clinical trials of natural nanomaterials in drug delivery

The translation of natural nanomaterials from laboratory research to clinical practice has been steadily increasing, with several promising case studies demonstrating their potential in drug delivery [122]. These studies provide critical insights into efficacy, safety, and formulation strategies, as well as guidance for future clinical translation.

### 7.1 Review of successful formulations in clinical trials

Several natural nanomaterial-based formulations have progressed to clinical trials, highlighting their therapeutic potential and biocompatibility. For instance, chitosan-based nanoparticles have been evaluated as oral delivery systems for vaccines and insulin, showing enhanced absorption, improved bioavailability, and minimal adverse effects. Similarly, alginate-based nanoparticles have been tested for oral and pulmonary delivery of drugs, demonstrating effective mucosal adhesion and controlled release in human studies [123].

Green-synthesized metallic nanoparticles, such as gold and silver nanoparticles, have also been explored for imaging-guided cancer therapy and targeted drug delivery, with early-phase trials reporting favorable safety profiles and enhanced tumor targeting. Additionally, lipid-polymer hybrid nanocarriers, combining natural polymers with lipids, have been assessed for delivery of chemotherapeutics and gene therapy agents, showing improved pharmacokinetics and reduced systemic toxicity in clinical settings [124].

Despite these promising developments, clinical translation remains limited due to several critical barriers identified in recent nanomedicine literature. First, there is a significant gap between preclinical success and clinical outcomes, as animal models often overestimate therapeutic efficacy due to differences in tumor physiology, immune response, and the enhanced permeability and retention (EPR) effect variability in humans [125]. Second, clinical trial limitations such as small sample sizes, short follow-up durations, and inconsistent endpoint standardization reduce the strength of evidence required for regulatory approval. Third, regulatory challenges remain a major obstacle, as agencies such as the FDA require extensive characterization of nanomaterial physicochemical properties, long-term toxicity, biodistribution, and batch-to-batch reproducibility before approval. Studies on lipid nanoparticle systems and albumin-based nanocarriers emphasize that although these platforms show strong translational promise, variability in manufacturing scale-up and biological performance remains a key bottleneck. Furthermore, long-term safety uncertainties, including immune response variability, nanoparticle clearance, and protein corona formation, continue to limit full clinical adoption of many nanocarrier systems despite encouraging early-phase trial results [126].

## 8 Emerging trends in natural nanomaterials for drug delivery

The field of natural nanomaterials for drug delivery is continuously evolving, with emerging trends focusing on multifunctional, hybrid, and precision therapeutic platforms. These innovations aim to overcome the limitations of conventional therapies and maximize the clinical potential of natural nanocarriers [127].

### 8.1 Nanoformulations for immunotherapy

One of the most promising trends is the application of natural nanomaterials in immunotherapy. Nanocarriers derived from natural polymers, lipids, or plant-derived compounds can deliver antigens, adjuvants, or immunomodulatory agents directly to immune cells, enhancing vaccine efficacy and immune responses. For example, chitosan-based nanoparticles have been explored for oral or intranasal vaccine delivery, showing improved mucosal immune activation [128]. Similarly, alginate and protein-based nanocarriers are being investigated for cancer immunotherapy, enabling precise delivery of tumor antigens and cytokines while minimizing systemic toxicity. These formulations not only enhance antigen stability and bioavailability but also allow controlled release, critical for sustained immune activation [129].

### 8.2 Hybrid nanomaterials combining natural and synthetic components

Another emerging trend is the development of hybrid nanomaterials, which integrate natural and synthetic components to combine the advantages of both. Natural polymers such as chitosan or alginate can be combined with synthetic polymers (e.g., PLGA) or inorganic nanoparticles (e.g., gold, silica) to enhance mechanical stability, drug loading capacity, and controlled release profiles [130]. Hybrid nanocarriers allow precise tuning of particle size, surface charge, and functionalization, enabling enhanced circulation time, targeted delivery, and stimuli-responsive behavior. These hybrid systems also provide versatility for encapsulating multiple therapeutic agents, imaging agents, or gene editing molecules within a single platform (Springer) [131].

### 8.3 Nanomaterial-based Co-delivery systems (drug + imaging agent)

The integration of co-delivery systems represents a major trend in theranostic applications, where nanocarriers simultaneously deliver therapeutic agents and imaging probes. Natural nanomaterials such as nanocellulose, chitosan nanoparticles, and liposomes can encapsulate chemotherapeutic drugs alongside fluorescent dyes, MRI contrast agents, or radioactive markers [132]. This dual functionality enables real-time monitoring of drug biodistribution, release kinetics, and therapeutic efficacy. Co-delivery systems also facilitate precision medicine approaches by allowing simultaneous treatment and diagnostic assessment, reducing treatment variability and improving patient outcomes [133].

## 9 Conclusion and future perspectives

Natural nanomaterials offer a biocompatible, biodegradable, and multifunctional platform for next generation drug delivery systems that can overcome many limitations of conventional therapies. A major limitation in current nanomedicine research is the lack of standardized quantitative benchmarking across nanocarrier systems. Drug loading efficiency, release kinetics, and pharmacokinetic enhancement vary widely across studies. For example, lipid nanoparticles can achieve up to ∼90% encapsulation efficiency, whereas polysaccharide systems typically range between 40 and 70% depending on formulation conditions. This variability limits direct comparison and clinical translation. By enhancing drug solubility, stability, targeting, and controlled release, these systems have the potential to significantly improve therapeutic outcomes while reducing side effects. Continued research, clinical validation, and collaborative efforts across disciplines are critical to realizing the full potential of natural nanomaterials in drug delivery. The authors conclude that the future development of nanocarrier-based drug delivery systems depends not only on continued material innovation but also on improving reproducibility, standardization of experimental and manufacturing processes, and strengthening translational pathways toward clinical application. Recent studies on biomaterial-, protein-, and lipid-based nanocarriers highlight that achieving consistent performance across formulations remains a key requirement for successful development and clinical translation. The field of natural nanomaterials for drug delivery is rapidly evolving, yet significant opportunities remain to enhance their clinical translation and therapeutic potential. Future research should focus on multidisciplinary integration, combining principles from chemistry, materials science, nanotechnology, pharmacology, and biomedical engineering. Such integration will allow the rational design of nanocarriers with optimized physicochemical properties, precise targeting capabilities, and improved biocompatibility. One of the critical priorities for future research is the development of scalable, environmentally friendly synthesis methods. Green synthesis approaches using plant extracts, microbial metabolites, or naturally derived polymers reduce environmental impact, avoid toxic reagents, and increase biocompatibility. Optimizing these methods for large-scale production without compromising nanoparticle uniformity, stability, or drug-loading efficiency is essential to facilitate industrial application. Research should also aim to establish standardized protocols that ensure reproducibility across batches and laboratories, thereby addressing one of the major barriers to clinical translation. Future investigations should incorporate robust characterization techniques to precisely define nanoparticle size, shape, surface chemistry, and drug release kinetics. Advanced imaging, spectroscopy, and computational modeling can provide detailed insight into nanoparticle–biological interactions, biodistribution, and pharmacokinetics. Additionally, the integration of advanced targeting strategies, including ligand functionalization, stimuli-responsive materials, and biomimetic coatings, will enhance site-specific delivery and reduce off-target effects. These strategies can be tailored to specific disease microenvironments, such as tumor acidity, enzymatic activity, or oxidative stress, for highly precise and responsive drug delivery. To advance natural nanomaterials toward clinical use, well-designed clinical studies are critical. Future work should emphasize preclinical studies that include comprehensive pharmacokinetics, biodistribution, toxicity, and immunogenicity assessments. Establishing standardized testing protocols will facilitate regulatory approval by ensuring that nanomaterial-based drug delivery systems meet safety, efficacy, and quality control requirements. Early engagement with regulatory agencies can help align experimental designs with clinical expectations, accelerating the translation of laboratory innovations into real-world therapeutic applications. Emerging technologies such as artificial intelligence (AI), machine learning, and bioinformatics can also play a pivotal role in the future of natural nanomaterials. AI-driven modeling can predict nanoparticle behavior, optimize formulations, and identify the most effective targeting strategies. Combined with high-throughput screening and microfluidic synthesis platforms, these technologies can significantly reduce development time, enhance precision, and improve the efficiency of designing multifunctional nanocarriers. Integration with personalized medicine approaches will further allow the customization of drug delivery systems to individual patient profiles, optimizing therapeutic outcomes. From a critical perspective, future progress in nanocarrier-based drug delivery will depend less on the discovery of entirely new materials and more on improving the reproducibility, scalability, and translational reliability of existing systems. Recent studies on lipid-, protein-, and biomaterial-based nanocarriers emphasize that consistent formulation performance and reliable biological behavior are key requirements for effective development and application in drug delivery systems. Improving standardization in preparation methods, characterization approaches, and biological evaluation models is therefore essential to ensure meaningful comparison across studies and enhance translational relevance. In addition, the incorporation of quantitative evaluation parameters such as drug loading efficiency, release behavior, and in vivo performance metrics is widely recognized in nanomedicine literature as necessary for strengthening evidence-based assessment of nanocarrier systems. Without addressing these core methodological and translational challenges, the successful transition of promising nanomaterials from experimental research to clinically approved therapies will remain limited.

## Ethics approval and consent to participate

Not applicable.

## Declaration of generative AI in scientific writing

Not applicable.

## Funding information

This research did not receive any specific grant from funding agencies in the public, commercial, or not-for-profit sectors.

## CRediT authorship contribution statement

**Great Iruoghene Edo:** Conceptualization, Data curation, Formal analysis, Funding acquisition, Investigation, Methodology, Project administration, Resources, Software, Supervision, Validation, Visualization, Writing – original draft, Writing – review & editing. **Ali B.M. Ali:** Validation, Visualization, Writing – original draft, Writing – review & editing. **Michael Chukwuma Okolie:** Validation, Visualization, Writing – original draft, Writing – review & editing. **Joshua Othuke Orogu:** Validation, Visualization, Writing – original draft, Writing – review & editing. **Kugbere Emumejaye:** Validation, Visualization, Writing – original draft, Writing – review & editing. **Ephraim Evi Alex Oghroro:** Validation, Visualization, Writing – original draft, Writing – review & editing. **Agatha Ngukuran Jikah:** Validation, Visualization, Writing – original draft, Writing – review & editing. **Emad Yousif:** Validation, Visualization, Writing – original draft, Writing – review & editing. **Ufuoma Augustina Igbuku:** Validation, Visualization, Writing – original draft, Writing – review & editing. **Joseph Oghenewogaga Owheruo:** Validation, Visualization, Writing – original draft, Writing – review & editing. **Arthur Efeoghene Athan Essaghah:** Validation, Visualization, Writing – original draft, Writing – review & editing. **Dina S. Ahmed:** Validation, Visualization, Writing – original draft, Writing – review & editing. **Raghda S. Makia:** Validation, Visualization, Writing – original draft, Writing – review & editing. **Huzaifa Umar:** Validation, Visualization, Writing – original draft, Writing – review & editing. **Ahmed A. Alamiery:** Validation, Visualization, Writing – original draft, Writing – review & editing. **Ibiyinka Agboola Fuwape:** Validation, Visualization, Writing – original draft, Writing – review & editing.

## Declaration of competing interest

The authors declare that they have no known competing financial interests or personal relationships that could have appeared to influence the work reported in this paper.

## Data Availability

All data will be made available upon reasonable request.
